# Nodule de sœur Marie-Josèphe

**DOI:** 10.11604/pamj.2019.33.228.19636

**Published:** 2019-07-18

**Authors:** Kawtar Kettani, Imane Chahid

**Affiliations:** 1 Département Pédiatrie 3 CHU Ibn Rochd, Casablanca, Maroc; 2 Département de Pédiartrie, Faculté de Médecine et de Pharmacie de Casablanca, Maroc

**Keywords:** Lymphome, nodule, nombril, Lymphoma, nodule, umbilicus

## Abstract

We here report the case of a 6-year old female patient with no pathological history, presenting with diffuse abdominal pain and abdominal distension evolving in the last 2 months. The patient had a deterioration in her general condition. Clinical examination showed distended abdomen, average abundance ascites, venous collateral circulation and multiple mobile hard abdominal masses measuring 3-4 cm in diameter at the level of the iliac fossa and of the left hypochondriac region associated with a well circumscribed, hard, rounded umbilical mass measuring 3cm in diameter lifting the skin with inflammatory signs (A). The remainder of the physical examination was normal. Thoraco-abdominal CT scan showed subcutaneous umbilical nodule (B), with homogeneous enhancement measuring 36x29x19mm, associated with mesenteric lymphadenopathy magmas, pelvic mass and extended jejunum thickening. Fine needle aspiration biopsy of the mass showed the presence of several naked nuclei and a few blasts. Biopsy showed Burkitt lymphoma. Therefore, the diagnosis of Burkitt lymphoma revealed by sister Marie-Josèphe nodule was retained and the patient underwent chemotherapy according the LMB01 protocol. Outcome was favorable, marked by the regression of the nodule and of the abdominal masses. Sister Mary Joseph nodule is an umbilical metastasis from a tumor (usually an abdomino pelvic tumor) mainly an adenocarcinoma, exceptionally a lymphoma. It is a rare sign. It accounts for 1-3% of all abdomino-pelvic neoplasms and it is associated with poor prognosis. It is essential to know that this nodule is a lesion secondary to solid tumor in order to avoid delayed management of the underlying neoplasm.

## Image en médecine

Nous rapportons le cas d'une patiente âgée de 6 ans, sans antécédents pathologiques, qui s'est présentée pour des douleurs abdominales diffuses et une distension abdominale évoluant depuis 2 mois dans un contexte d'altération de l'état général. L'examen clinique a trouvé un abdomen distendu, une ascite de moyenne abondance, une circulation veineuse collatérale et de multiples masses abdominales mobiles, de consistances dures, de 3-4 cm de diamètres au niveau de la fosse iliaque et de l'hypochondre gauche avec la présence d'une masse ombilicale arrondie, bien circonscrite, dure, de 3cm de diamètre soulevant la peau avec des signes inflammatoires en regard (A). Le reste de l'examen clinique était normal. Le scanner thoraco-abdominal a mis en évidence un nodule ombilical sous cutané (B), à rehaussement homogène mesurant 36x29x19mm, associé à un magma d'adénopathies mésentériques, une masse pelvienne et un épaississement étendu du jéjunum. La cytoponction de la masse a objectivé la présence de plusieurs noyaux nus et quelques blastes et la biopsie était en faveur d'un lymphome de Burkitt. Ainsi, le diagnostic de lymphome de Burkitt révélé par un nodule de Sœur Marie-Josèphe a été retenu et la patiente a été mise sous chimiothérapie selon le protocole LMB01. L'évolution a été favorable, marquée par la régression du nodule et des masses abdominales. Le nodule de Sœur Marie-Josèphe est une métastase ombilicale d'une tumeur le plus souvent abdomino-pelvienne essentiellement un adénocarcinome, exceptionnellement un lymphome. C'est un signe rare dont l'incidence est de 1-3% de toutes les néoplasies abdomino-pelviennes, avec un pronostic péjoratif. Ce nodule mérite d'être connu comme lésion secondaire d'une tumeur solide afin d'éviter un retard de prise en charge de la néoplasie sous-jacente.

**Figure 1:**
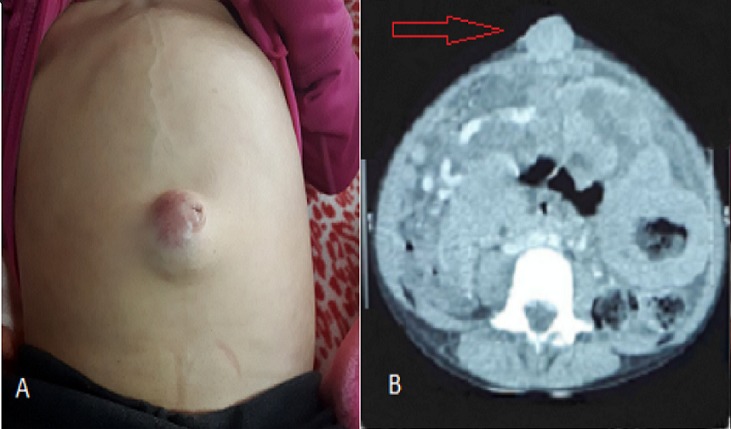
A) nodule de sœur Marie-Josèphe; B) aspect scannographique du nodule

